# Fibrin Sealants and Axillary Lymphatic Morbidity: A Systematic Review and Meta-Analysis of 23 Clinical Randomized Trials

**DOI:** 10.3390/cancers13092056

**Published:** 2021-04-24

**Authors:** Maria Luisa Gasparri, Thorsten Kuehn, Ilary Ruscito, Veronica Zuber, Rosa Di Micco, Ilaria Galiano, Siobana C. Navarro Quinones, Letizia Santurro, Francesca Di Vittorio, Francesco Meani, Valerio Bassi, Nina Ditsch, Michael D. Mueller, Filippo Bellati, Donatella Caserta, Andrea Papadia, Oreste D. Gentilini

**Affiliations:** 1 Department of Gynecology and Obstetrics, Ospedale Regionale di Lugano EOC, via Tesserete 46, 6900 Lugano, Switzerland; marialuisa.gasparri@eoc.ch (M.L.G.); francesco.meani@eoc.ch (F.M.); valerio.bassi@eoc.ch (V.B.); 2 Faculty of Biomedical Sciences, Università della Svizzera Italiana (USI), via Giuseppe Buffi 13, 6900 Lugano, Switzerland; 3 Interdisciplinary Breast Center, Department of Gynecology and Obstetrics, Klinikum Esslingen, 73730 Neckar, Germany; t.kuehn@klinikum-esslingen.de; 4 Gynecology Division, Department of Medical and Surgical Sciences and Translational Medicine, Sant’Andrea University Hospital, Sapienza University of Rome, via di Grottarossa 1035, 00189 Rome, Italy; ilary.ruscito@uniroma1.it (I.R.); filippo.bellati@uniroma1.it (F.B.); donatella.caserta@uniroma1.it (D.C.); 5 Breast Surgery Unit, Department of Surgery, San Raffaele University Hospital, via Olgettina 60, 20132 Milan, Italy; zuber.veronica@hsr.it (V.Z.); dimicco.rosa@hsr.it (R.D.M.); galiano.ilaria@hsr.it (I.G.); santurro.letizia@hsr.it (L.S.); divittorio.francesca@hsr.it (F.D.V.); gentilini.oreste@hsr.it (O.D.G.); 6 Department of Clinical Medicine and Surgery, University of Naples Federico II, 80138 Naples, Italy; 7 Emory University, Atlanta, GA 30322, USA; scnavar@emory.edu; 8 Department of Gynecology and Obstetrics, University Hospital of Augsburg, Stenglinstraße 2, 86156 Augsburg, Germany; nina.ditsch@uk-augsburg.de; 9 Department of Obstetrics and Gynecology, University Hospital of Bern, Friedbühlstrasse 19, 3010 Bern, Switzerland; michel.mueller@insel.ch

**Keywords:** axillary lymphadenectomy, breast cancer, fibrin sealant, lymphatic morbidity, lymphocele

## Abstract

**Simple Summary:**

Axillary dissection is a highly mobile procedure with severe lymphatic consequences. The off-label application of fibrin sealants in the axilla, with the sole aim to eliminate dead space and to provoke sealing of the disrupted lymphatic vessels at the end of axillary dissection, is an experimental procedure to reduce lymphatic morbidity. The aim of our systematic review and meta-analysis is to investigate the effects of fibrin sealants on lymphatic morbidity after axillary dissection. Our results show that this experimental procedure is able to decrease the total axillary drainage output, the number of days before the axillary drainage is removed, and the length of hospital stay. However, no effects on the occurrence rate of axillary lymphocele or on the surgical site complications rate were demonstrated

**Abstract:**

Background: use of fibrin sealants following pelvic, paraaortic, and inguinal lymphadenectomy may reduce lymphatic morbidity. The aim of this meta-analysis is to evaluate if this finding applies to the axillary lymphadenectomy. Methods: randomized trials evaluating the efficacy of fibrin sealants in reducing axillary lymphatic complications were included. Lymphocele, drainage output, surgical-site complications, and hospital stay were considered as outcomes. Results: twenty-three randomized studies, including patients undergoing axillary lymphadenectomy for breast cancer, melanoma, and Hodgkin’s disease, were included. Fibrin sealants did not affect axillary lymphocele incidence nor the surgical site complications. Drainage output, days with drainage, and hospital stay were reduced when fibrin sealants were applied (*p* < 0.0001, *p* < 0.005, *p* = 0.008). Conclusion: fibrin sealants after axillary dissection reduce the total axillary drainage output, the duration of drainage, and the hospital stay. No effects on the incidence of postoperative lymphocele and surgical site complications rate are found.

## 1. Introduction

Axillary lymph node dissection (ALND) was historically proposed as an integral part of the treatment of breast cancer, melanoma, and Hodgkin’s disease. In several conditions, ALND was replaced by sentinel lymph node biopsy (SLNB) as axillary staging procedure in breast cancer [1,2,3]. Full axillary lymph node dissection is restricted to node-positive cases as a therapeutic approach. This procedure can result in significant postoperative complications. One of the most common problem following full lymphadenectomy is the lymphocele. Lymphocele is a collection of fluid deriving from the excess lymphatic drainage following the removal of lymph nodes. Lymphoceles can easily be diagnosed by a clinical examination or by ultrasound. This condition may result in pain, repeated aspirations, infections, and could delay local healing and adjuvant treatments. Currently, insertion of suction drains at the time of ALND is the most common surgical method used to reduce lymphocele formation after ALND and subsequent wound complications. Drains were demonstrated to reduce the incidence of axillary lymphocele; however, they require ongoing surveillance and maintenance, entail a certain degree of discomfort, and limit physical activity [4,5]. The criteria for drain removal vary according to institutions and surgical practices (Table 1). Recently, the tendency has been to keep it in as short a time as possible, and in some centers, the possibility of not draining the axilla with postoperative drains following ALND is currently adopted. Unfortunately, even when drains are applied, the percentage of lymphocele formation is not negligible. Therefore, several additional or alternative strategies in the attempt to reduce the rate of lymphoceles and lymphatic complications occurring after a systematic lymphadenectomy were proposed. They include the closure of dead space by different means, delay of exercise, the spraying of tetracycline, the use of external compression dressing, and the use of modified dissection tools such as the harmonic scalpel. Nonetheless, none of these procedures improved the results [6,7,8]. Fibrin sealants, in the form of glue or patches, were approved as hemostatic agents across a wide range of settings, ranging from breast surgery to orthopedic surgery [9]. So far, the adoption of fibrin sealants following ALND with different aims is considered an off-label use. The prophylactic use of fibrin sealant patches has been investigated and it is still under evaluation in different fields, such as axillary, pelvic, para-aortic, and inguinal dissection. When a multi-disciplinary analysis was performed, encouraging effects were found [10]. When fibrin sealants are off-labeling, adopted to reduce the postoperative morbidity after lymph node dissection, they are applied on raw wounded areas underneath skin flaps at the end of the ALND. The rationale of this application is that fibrin seals might potentially reduce lymphocele formation by adhering previously elevated skin flaps; thus, eliminating dead space and working on lymphatic vessels in a similar way as for blood vessels [11,12,13]. The aim of this meta-analysis is to investigate the effectiveness of fibrin sealants on the incidence of lymphocele and related lymphatic morbidity after ALND, as well as to evaluate if the same results achieved in other settings, such as pelvis, inguinal area and upper abdomen, can be applied in the axilla.

## 2. Materials and Methods

### 2.1. Data Selection

This meta-analysis was performed based on the Preferred Reporting Items for Systematic reviews and Meta-Analyses (PRISMA) statement. The methodology steps from the design of the study to the manuscript writing are reported in Table A1. A systematic literature search was performed on September 2019 and repeated on August 2020, from inception. Two authors (I.G. and I.R.) independently performed the review of the literature and the studies’ selection, if there was a conflict, a third author (M.L.G.) solved it. Data were identified using the electronic databases PubMed, MEDLINE, and Scopus by searching for the terms “axillary lymphadenectomy” or “axillary dissection” and “seroma” or “lymphocele” and “fibrin sealant”. When comparing studies retrieved with the terms “axillary lymphadenectomy” and “axillary dissection”, only duplicates and/or studies out of topics were found, searching by the term “axillary dissection”; therefore, this term was excluded by the terms used in the process selection. All original reports evaluating the use of fibrin sealants on axillary lymphadenectomy and related lymphatic complications were examined for inclusion. Papers regarding various axillary lymphadenectomies performed for different malignant diseases were all evaluated for inclusion, most of them referred to breast cancer and melanoma. No study properly defined the term “lymphocele”; therefore, both seroma and lymphocele were equally adopted to define postoperative lymphatic complication consisting in a clinically or radiological finding of fluid collection after ALND. In order not to miss any other potential study, all references of original reports and reviews already published were also reviewed. All randomized controlled studies evaluating the efficacy of different fibrin sealants devices were included in the meta-analysis, including liquid (glue/gel) and non-liquid (patches) forms. The sealants could be fibrin/fibrinogen based, mixed (or not mixed) with thrombin. Only clinical randomized trials were considered in the analysis. All studies without any publication time intervals were included. No language restriction was applied in the literature search. Review articles, case reports, and letters were excluded. The following data were considered in the meta-analysis, adopted for the analysis, and summarized in the table of the included studies: first author’s information and publication year, sample size of cases and controls, type of cancer (breast cancer, melanoma, lymphoma), type of breast surgery (null, lumpectomy, quadrantectomy, mastectomy), level of axillary dissection (I, II, III), number of lymph nodes removed, number of lymph nodes involvement, type of sealant device (patch, gel, glue), quantity of the sealant device (mL for gel and glue, number of device for patches), percentages of patients undergoing neo-adjuvant chemotherapy before surgery, criteria for drainage removal, number of drainage days, incidence of postoperative axillary lymphocele, axillary drainage output (mL), days of hospital stay, surgical site complications including surgical site infections, cellulitis, fever, wound dehiscence, local inflammation, and skin necrosis among patients undergoing axillary lymphadenectomy, with and without the application of a sealant device at the end of the procedure, for the sole purpose of preventing axillary lymphadenectomy related complications. Data from surgical-site complications were pooled together and each complication was considered as an event.

### 2.2. Outcomes

The outcomes considered for patients undergoing axillary lymphadenectomy with or without the application of sealant devices at the end of the procedure were the following: primary outcome: incidence of postoperative axillary lymphocele; secondary outcomes: volume of lymph drained, number of drainage days, axillary drainage output, days of hospital stay, and surgical site complications.

### 2.3. Statistical Analysis

Dichotomous and continuous data were analyzed using Review Manager 5.3 (http://www.cochrane.org, accessed on 1 August 2020). Data for continuous outcomes were not included in the meta-analysis if standard deviations or standard errors of mean were not reported or could not be calculated. Risk of developing lymphatic morbidity in patients undergoing ALND with and without the use of sealant devices was stratified by studies and the pooled odds ratio (OR) was calculated using a fixed- or a random-effects model. Differences in the considered outcomes were stratified by studies, and the weighted mean differences (WMDs) were calculated using a fixed- or a random-effects model. A χ^2^ test for heterogeneity among proportions was performed to verify the presence of statistical heterogeneity between studies. Graphical representation of each study and pooled analysis were displayed by forest plots. The weight of each study was graphically represented as a square of different size. Confidence intervals (CIs) for each study were symbolized as a horizontal line passing through the square. The pooled OR or WMD was displayed as a lozenge in the forest plot and its size corresponded to the 95% CI of the OR or WMD. A *p* value ≤ 0.05 was defined as significant.

## 3. Results

**Figure 1:**
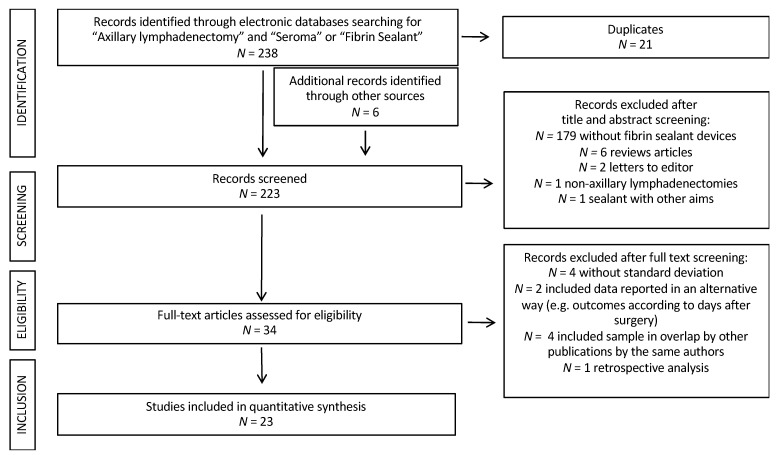
PRISMA flow diagram on the meta-analysis process.

Overall, 238 studies were retrieved through the literature search and six additional records were identified through reference lists. Twenty-one studies were removed as duplicates. One-hundred and eighty-nine further records were excluded after title and abstract evaluation because 179 papers did not adopt fibrin sealant devices, 8 papers were published as reviews or letters to the editor only, one paper was conducted on patients undergoing pelvic, paraaortic and inguinal lymphadenectomies for gynecological malignancies, and one paper investigated the efficacy of fibrin sealant for different outcomes. One study was excluded from the analysis due to its retrospective nature [13]. At the end of the selection process, 23/223 (10.3%) screened studies met the inclusion criteria [14,15,16,17,18,19,20,21,22,23,24,25,26,27,28,29,30,31,32,33,34,35,36]. Figure 1 shows the PRISMA flow chart outlining the process of evidence acquisition. The flow chart specifies the number of studies identified, screened, included, and excluded, as well as the reasons for exclusions. Overall, 1988 patients undergoing ALND were included in the analysis. Among them, 1884 (94.8%) patients had breast cancer; the remaining 103 and 1 patients were affected by melanoma and other cancers, and Hodgkin’s disease, respectively. In one of the series, one patient with Hodgkin’s disease underwent an axillary dissection [27]. The authors do not comment on the reason why they subjected the patient to this procedure. Nine hundred and thirty-five (47%) patients used the fibrin devices, with the scope of lymphatic complication reduction. Patients who required fibrin sealants for hemostatic reasons were excluded from the analysis. Postoperatively, suction drains were placed in every case. The characteristics of the studies and the surgical data are listed in Table 1 and Table 2, respectively. Risk of bias assessment is reported in Table 3.

### 3.1. Axillary Lymphocele

**Figure 2:**
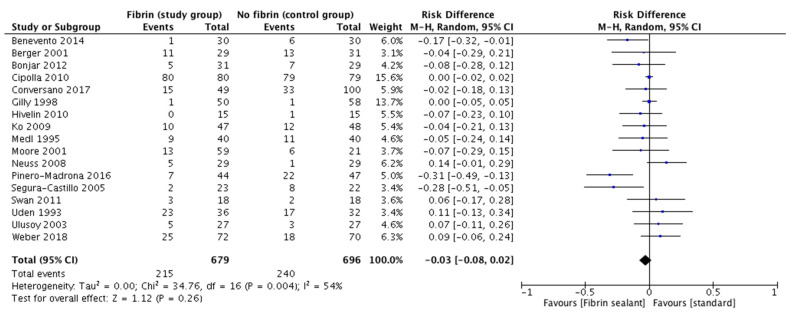
Axillary lymphocele.

**Figure A1:**
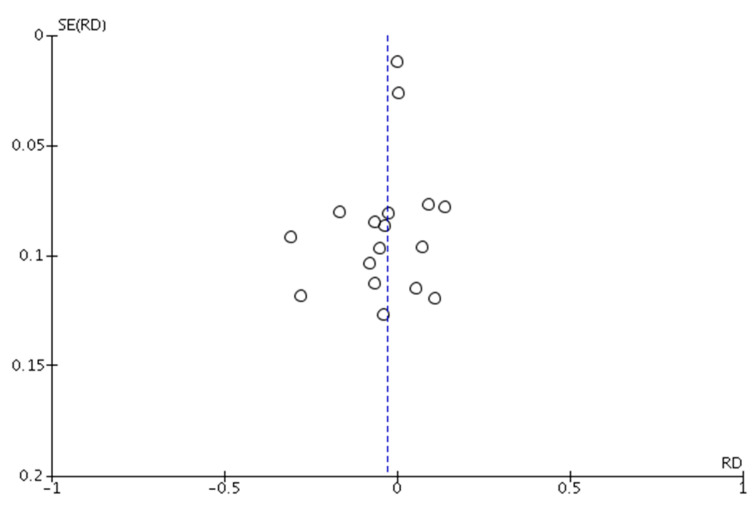
Funnel Plot: Axillary Lymphocele.

Seventeen trials reporting data on incidence of lymphocele could be used for the comparison. The pooled analysis showed no significant decrease in incidence of postoperative lymphocele when sealant devices were used (odds ratio: −0.03 [95%CI. −0.08, −0.02]; *p* 0.26, random effects model) (Figure 2). A funnel plot is available in Figure A1.

### 3.2. Axillary Drainage Output

**Figure 3:**
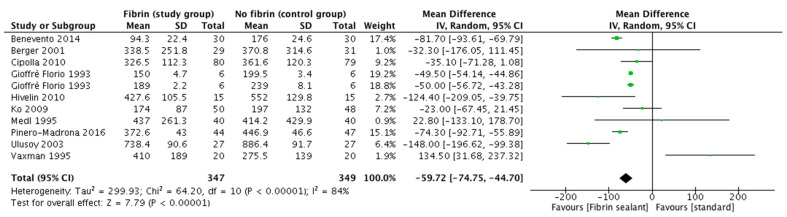
Axillary drainage output. The value reported for the axillary drainage output refers to the mean total drainage volume, in mL, +/− standard deviation.

Eleven trials reported data on axillary drainage output could be used for the comparison. The pooled analysis showed a significant decrease in the drainage output of those patients in whom a sealant device was used for this purpose (odds ratio: −59.72 [95%CI. −74.75, −44.7]; *p* < 0.0001, random effects model) (Figure 3).

### 3.3. Days of Hospital Stay

**Figure 4:**
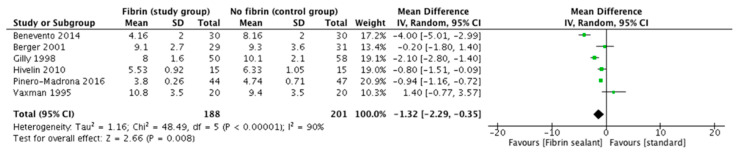
Days of hospital stay.

Six trials reporting data on hospital stay could be used for the comparison. The pooled analysis showed a significant difference in incidence of days of hospital stay when sealant devices were used for this purpose (odds ratio: −1.32 [95%CI. −2.2, 0.3]; *p* 0.008, random effects model) (Figure 4).

### 3.4. Surgical Site Complications

**Figure 5:**
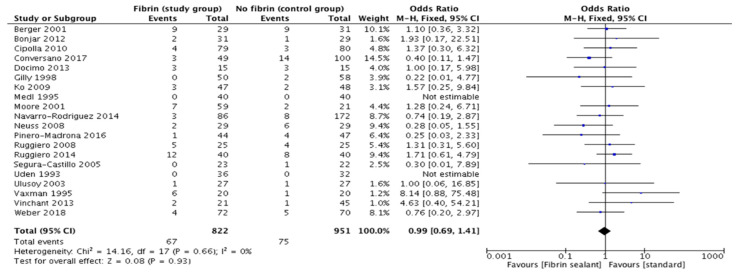
Surgical site complications.

Nineteen trials reporting surgical site complications could be used for the comparison. The pooled analysis showed no significant difference in surgical site complications when sealant devices were used for this purpose (odds ratio: 0.99 [95%CI. 0.69, 1.41]; *p* 0.93, fixed effects model) (Figure 5).

### 3.5. Number of Days before Removal of Axillary Drainage

**Figure 6:**
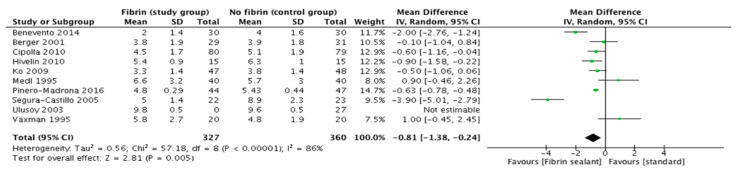
Number of days before removal of axillary drainage.

Ten trials reporting data on the length of drain maintenance could be used for the comparison. The pooled analysis showed a significant decrease in the number of days before axillary drainage is removed in those patients in whom a sealant device was used for this purpose (Odds Ratio: −0.8 [95%CI. −1.38, −0.24]; *p* = 0.005, random effects model) (Figure 6).

## 4. Discussion

Following ALND, a lymphocele may occur in the dead space of the axilla with an incidence of up to 85% [37,38]. Lymphocele consists in an abnormal fluid collection, causing significant morbidity, delayed wound healing, and quality of life. It can be associated with a delay in the beginning of adjuvant treatment and, consequently, affect the oncologic outcome [39,40]. Sometimes, additional surgical procedures are required in case of long-standing persistent lymphocele [41]. The most common method to reduce the incidence of the lymphocele is the use of postoperative drainage. Several randomized trials have aimed to replace the drains by reducing seroma formation in different ways, but with inconsistent results [6,42,43,44]. Advanced hemostasis was also proposed as a lymphocele prevention tool after ALND in breast cancer patients [45]. Fibrin sealants were proposed to reduce the lymphatic morbidity following various surgeries, such as cardiovascular surgery, prostate surgery, and rhytidectomy [46,47,48]. The rationale of its use is to reduce the dead space of the cavity by obliterating the potential space between tissue layers and to promote tissue healing and lymphatic vessels closure. In patients undergoing mastectomy and sentinel lymph node biopsy due to invasive breast cancer or ductal carcinoma in situ, the use of fibrin sealant reduces the postoperative lymphocele aspiration [49] and the drain-free breast surgery [50]. Very recently, some authors suggested that the use of fibrin sealants after breast surgery gave surgeons the confidence to opt for a drain-free technique, thus enhancing the day-case surgery rate [51]. When fibrin sealants are applied to prevent lymphatic morbidity following lymphadenectomies, a recent meta-analysis showed that fibrin sealant devices reduced lymphatic drainage following pelvic, para-aortic, inguinal, and axillary lymphadenectomies for gynecological malignancies and breast cancer [10]. The same investigation in the sole axillary area achieved conflicting results. When fibrin glue instillation under skin flaps was used to prevent lymphocele related morbidity following breast and axillary surgery, insufficient evidence to clearly assess the benefit on lymphocele rates and volume, complications, and length of hospital stay was achieved [52]. A more pronounced benefit was identified when non-liquid fibrin sealant devices (patches) were adopted in the same setting [24,26,27]. However, in a recent phase III clinical randomized trial, the application of fibrin sealant patches after ALND did not significantly reduce the total volume of axillary drainage. Moreover, the length of hospital stay was not increased in this group of patients [36]. In our meta-analysis, the use of a sealant device in patients undergoing ALND did not affect the incidence of postoperative lymphocele, the length of hospital stay, and the surgical site complications, neither significantly nor relevantly, despite the significant reduction observed in the total axillary drainage output and the number of days before the axillary drainage was removed. Our data reveals that the use of a fibrin sealant device after axillary dissection does not affect the incidence of postoperative lymphocele and surgical site complications; however, it reduces the total axillary drainage output, the days before the axillary drainage is removed, and the hospital stay. The strength of our meta-analysis is the exclusive selection of clinical randomized trials. Furthermore, compared to previous pool analyses considering multiple sites of lymphadenectomy, we only considered the axilla as surgical site. However, our results may be accompanied by some limitations. A potential limit is the heterogeneity arising from the single studies, included in the analysis. As for instance, no restrictions about surgical procedures and/or surgical outcomes (e.g., number of removed lymph nodes and level of ALND) are always applied in the studies, as reported in Table 2. However, since only randomized trials were included in the analysis, it is plausible to consider that the randomization reduced the risk of bias within each study. Nevertheless, the pooled analysis might be biased anyway. In order to quantify this potential bias, a risk of bias assessment was performed for each included study, as reported in Table 3. In particular, the type and stage of diseases and the surgical procedures associated to ALND in the selected studies differed and it may have affected the results. For instance, in the case of mastectomy, breast and axillary cavities communicate and therefore the drain output might be higher as compared to ALND associated with quadrantectomy or ALND alone. Similarly, in our analysis we included also studies in which level III axillary dissection was performed. It is intuitive to consider patients undergoing level III lymphadenectomy at higher risk of lymphatic complications. Given the decreasing rate of level III axillary dissection in several institutions, it would be interesting, in the future, to investigate two different subsets of patients, including those at higher and lower risk of lymphatic morbidity, separately. Secondly, all the sealant devices included in the analysis were fibrin sealants, with or without thrombin, in different quantity and forms (glue and patch). Furthermore, some studies included surgical procedures performed after neo-adjuvant chemotherapy (Table 1). Finally, the authors adopted different criteria for the drainage removal; therefore, the difference in length of drainage maintenance should be interpreted carefully. Similarly, the results regarding the days of hospital stay should be interpreted carefully due to the wide variability of the hospitalization criteria in this setting. As, for instance, in several institutions, according to internal policy, the presence of drainage is a reason for hospitalization whereas in others the drainage has a domiciliary use. Furthermore, in some institutions, the diagnosis-related reimbursement system discourages early discharge after breast surgery, thus determining a wide range on the length of hospital stay. In addition, the time window in which the included studies on this topic were published is wide. As compared to older practices, more recent policies support the day surgery or discharge in the morning following the day of breast surgery, irrespective of the axillary procedure. Therefore, data on hospital stay might be a reflection of older studies and conclusion about the length of hospital stay should be considered carefully and interpreted just as an indirect indicator. Similarly, the performance of level III dissections is also an indication of some older studies and, thus, may not reflect or be applicable to current practice. Finally, it is not clear, whether the number of positive lymph nodes may affect the post-operative lymphatic morbidity. Despite this, none of the studies on this topic performed a subgroup analysis differentiating the status of lymph node involvement in the control and experimental group, respectively. Similarly, not only the treatment the patients underwent, but also the stage of disease might have an impact on the post-operative lymphatic morbidity. A cost effectiveness analysis on the use of sealant devices to prevent lymphadenectomy related complications was not the aim of the study. An ongoing trial from the MD Anderson Cancer Center is currently active to determine whether the use of fibrin sealant applied to axillary soft tissues following ALND for melanoma can result in reduced lymphatic morbidity, and to assess patient-evaluation of outcome by performing a cost benefit analysis using a willingness-to-pay model [53]. The results of this study will probably provide useful additional evidence on the topic. Similarly, recent evidence showed a significant decrease in the rate of postoperative percutaneous aspiration of lymphocele in those patients in whom a sealant patch was used for reducing lymphatic morbidity [10]. However, this recent analysis was not applied to the sole ALND but it included pelvic, para-aortic, and inguinal lymphadenectomies. Given the current evidence and the lack of cost effectiveness analysis, we recommend a prudent use of fibrin sealants after ALND with the aim to reduce lymphatic morbidity. It might be reasonable to use fibrin sealants in those cases in which a high lymphatic output is expected such as in patients undergoing both, mastectomy and ALND (leading to a large surgical surface), high axillary tumor burden or extensive axillary surgery (e.g., including level III). In some studies, the use of hemostatic patches has been associated with an increased risk of infections [54]. It has been hypothesized that the reason for the increased risk of reported infections was rather related to the bloodier surgical procedure requiring the adoption of the patch than on the patch itself [55]. Consequently, no increased risk of infections is expected when fibrin sealants are used to reduce the lymphatic output after ALND. In fact, this was not the case in the studies reviewed in our meta-analysis.

## 5. Conclusions

The use of fibrin sealant devices after ALND, with the sole aim of reducing lymphatic morbidity, is able to decrease the total axillary drainage output, the number of days before the axillary drainage is removed, and the length of hospital stay. However, no effects on the occurrence of axillary lymphocele or on the surgical site complications rate were achieved. These results warrant further evidence before they can be considered conclusive. It would be useful to compare the relative costs of fibrin sealant, and its outcome, in order to determine if its use justifies its costs.

## Figures and Tables

**Table 1:** Characteristics of the clinical randomized trials.

Author (Ref)	Tumor Type	Pts Study Group/Tot	Fibrin Device	Quantity	NACT *N* (%)	Criteria for Drainage Removal (mL/24 h)
BENEVENTO 2014 [14]	Breast cancer	30/60	Tisseel glue	4 mL	NR	≤30
BERGER 2001 [15]	Breast cancer	30/60	TachoSil patch	1,5 patches	Excluded	≤70
CIPOLLA 2010 [16]	Breast cancer	80/159	Glue	2 mL	Excluded	≤80
CONVERSANO 2017 [17]	Breast cancer	49/149	Tisseel glue	4 mL	22 (44.9%) study group 23 (23%) control group	≤50
DOCIMO 2013 [18]	Breast cancer	15/30	Tisseel glue	2 mL	NR	≤100
GILLY 1994 [19]	Breast cancer	50/108	Tisseel glue	2 mL	Excluded	After 6 days
HIVELIN 2011 [20]	Breast cancer	15/30	Tisseel glue	5 mL	NR	≤30
KO 2009 [21]	Breast cancer	50/100	glue	2 mL	Excluded	≤30
MEDL 1995 [22]	Breast cancer	67/142	TachoSil patch	3 patches	21 (29.2%) study group 21 (30%) control group	≤30
MIRI BONJAR 2012 [23]	Breast cancer	31/60	glue	2 mL	NR	≤30
MOORE 2001 [24]	Breast cancer	59/80	glue	4 mL, 8 mL, 16 mL	NR	≤40
NAVARRO-RODRIGUEZ 2014 [25]	Breast cancer	86/258	TachoSil patch	2 patches	16 (18.6%) study group 32 (28.6%) C	≤50
NEUSS 2008 [26]	Melanoma	29/58	Tisseel glue	2 mL	NR	≤50
PINERO-MADRONA 2016 [27]	Melanoma, Breast cancer, Hodgkin’s disease	44/91	TachoSil patch	3 patches	24	≤50
RUGGIERO 2008 [28]	Breast cancer	25/50	Glue + patch	2 mL	NR	≤100
RUGGIERO 2009 [29]	Breast cancer	45/90	Tisseel glue	2 mL	NR	≤100
RUGGIERO 2014 [30]	Breast cancer	40/80	Tisseel glue	2 mL	NR	≤100
SEGURA-CASTILLO 2005 [31]	Breast cancer	22/43	Quixil gel	10 mL	11 (20%) study group 10 (20%) control group	≤50
SWAN 2011 [32]	Melanoma Others	18/36	Tisseel glue	4 mL	NR	≤30
UDEN 1992 [33]	Breast cancer	36/68	Tisseel glue	2 mL	NR	≤100
ULUSOY 2003 [34]	Breast cancer	27/54	Tisseel glue	4 mL	Excluded	≤20
VAXMAN 1995 [35]	Breast cancer	20/40	Tisseel glue	5 mL	NR	≤10
WEBER 2018 [36]	Breast cancer	67/142	TachoSil patch	3 patches	21 (29.2%) study group 21 (30%) control group	≤30

Acronyms: Pts: patients; NACT: neoadjuvant chemotherapy; NR: not reported.

**Table 2:** Surgical data.

Author (ref)	Breast Surgery	Level of Dissection	N. Nodes Removed (Median)	N. Positive Nodes (Median)	Pts with Positive Nodes
BENEVENTO 2014 [14]	Quadrantectomy, mastectomy	III	23.7 study group 24 control group	NR	NR
BERGER 2001 [15]	Lumpectomy, mastectomy	II	NR	NR	NR
CIPOLLA 2010 [16]	Quadrantectomy, mastectomy	III	16.6 study group 17.4 control group	NR	46 study group 3 control group
CONVERSANO 2017 [17]	Quadrantectomy or ALND only	II	14.5 study group 14 control group	1 study group 1 control group	NR
DOCIMO 2013 [18]	Quadrantectomy, mastectomy	NR	NR	NR	NR
GILLY 1994 [19]	Modified radical mastectomy, sector mastectomy	III	10.6 study group 0.8 control group	1.9 study group 1.9 control group	NR
HIVELIN 2011 [20]	Mastectomy	NR	NR	NR	NR
KO 2009 [21]	Quadrantectomy	I-III	12.6 study group 12.5 control group	0.8 study group 0.8 control group	NR
MEDL 1995 [22]	Quadrantectomy, mastectomy	NR	12.8 study group 3.9 control group	4.2study group; 4.4 control group	NR
MIRI BONJAR 2012 [23]	Quadrantectomy, mastectomy	NR	14.7study group 4.2 control group	1.2 study group 2.5 control group	NR
MOORE 2001 [24]	Lumpectomy, mastectomy	NR	NR	NR	NR
NAVARRO-RODRIGUEZ 2014 [25]	Quadrantectomy, mastectomy	III	14.95 study group 15.66 control group	NR	64 Study group 102 control group
NEUSS 2008 [26]	Null	III	16 study group ; 15 control group	3study group 3 control group	NR
PIERO MADRONA [27]	NR	II; III	16.59 study group 18.36 control group	NR	NR
RUGGIERO 2008 [28]	Quadrantectomy, mastectomy	I; II	18.1 study group 18.7 control group	12 study group 13 control group	NR
RUGGIERO 2009 [29]	Quadrantectomy, mastectomy	I; II	NR	NR	NR
RUGGIERO 2014 [30]	Quadrantectomy, mastectomy	I; II	NR	NR	NR
SEGURA-CASTILLO 2005 [31]	Mastectomy	III	NR	NR	NR
SWAN 2011 [32]	NR	III	18 study group 12.5 control group	NR	NR
UDEN 1992 [33]	Mastectomy	III	NR	NR	NR
ULUSOY 2003 [34]	Mastectomy	III	NR	5.7 study group 2.66 control group	NR
VAXMAN 1995 [35]	Quadrantectomy, Patey’s mastectomy	NR	10.8 study group 9.3 control group	2.9 study group 2.1 control group	11 study group 11 control group
WEBER 2018 [36]	Quadrantectomy	II; III	16 study group 18.5 control group	2 study group 3 control group	NR

Acronyms: NR: not reported.

**Table 3:** Risk of bias assessment.

	Type of Bias	Selection Bias	Performance Bias	Detection Bias	Attrition Bias
Study (Ref)					
BENEVENTO 2014 [14]		Low risk	Intermediate risk	Low risk	Intermediate risk
BERGER 2001 [15]		Low risk	Intermediate risk	Intermediate risk	Intermediate risk
CIPOLLA 2010 [16]		Low risk	Intermediate risk	Low risk	Intermediate risk
CONVERSANO 2017 [17]		Intermediate risk	Intermediate risk	Intermediate risk	Intermediate risk
DOCIMO 2013 [18]		NA	Intermediate risk	Low risk	Intermediate risk
GILLY 1994 [19]		Low risk	High risk	Low risk	Intermediate risk
HIVELIN 2011 [20]		NA	NA	NA	NA
KO 2009 [21]		High risk	Low risk	Low risk	Low risk
MEDL 1995 [22]		High risk	High risk	Intermediate risk	Intermediate risk
MIRI BONJAR 2012 [23]		High risk	Low risk	Low risk	Intermediate risk
MOORE 2001 [24]		Low risk	Low risk	Low risk	Low risk
NAVARRO-RODRIGUEZ 2014 [25]		Low risk	Intermediate risk	Low risk	Intermediate risk
NEUSS 2008 [26]		NA	Intermediate risk	Low risk	Intermediate risk
PINERO MADRONA [27]		NA	Low risk	Low risk	Low risk
RUGGIERO 2008 [28]		Intermediate risk	Intermediate risk	Low risk	Intermediate risk
RUGGIERO 2009 [29]		Intermediate risk	Low risk	Low risk	Low risk
RUGGIERO 2014 [30]		Intermediate risk	Low risk	Intermediate risk	Low risk
SEGURA-CASTILLO 2005 [31]		Low risk	Intermediate risk	Low risk	High risk
SWAN 2011 [32]		NA	Low risk	Low risk	Low risk
UDEN 1992 [33]		Low risk	Intermediate risk	Intermediate risk	Intermediate risk
ULUSOY 2003 [34]		Low risk	Low risk	High risk	Low risk
VAXMAN 1995 [35]		NA	Low risk	Low risk	Low risk
WEBER 2018 [36]		Low risk	Low risk	Low risk	Low risk

Type of bias definition: selection bias: potential difference in baseline characteristics between the two groups; performance bias: potential difference in treatment, other than intervention, between the two groups; detection bias: potential difference between the two groups in how outcomes are determined; attrition bias: potential difference in loss to follow up/withdrawals between the two groups.

## Data Availability

The data presented in this study are available in the present manuscript.

## Appendix A

**Table A1:** Methodology Steps.

TASK	DESCRIPTION	CLASSIFICATION
Formulate question	Question: Does the off-label use of fibrin sealant affect lymphatic morbidity after axillary dissection?	Step I: preparation
Check for previous systematic review (SR)	Similar SRs, but in different settings were identified and commented in the discussion.	Step I: preparation
Protocol drafting	An objective and reproducible methodology was designed and reported in the “methods” paragraph of the manuscript.	Step I: preparation
Search strategy definition	Data were identified using the electronic databases PubMed, MEDLINE, and Scopus and strict inclusion criteria were defined.	Step I: preparation
Literature search	238 citations, even if many irrelevant ones included at the first literature screening.	Step II: retrieval
De-duplicate	Twenty-one identical citations were excluded.	Step II: retrieval
Screen abstract	Based on titles and abstract, the irrelevant trials were definitely removed.	Step III: appraisal
Obtain full text	All the remaining studies were downloaded. When download was not allowed, a copy was requested from author or alternative library.	Step II–III: retrieval and appraisal
Screen full text	Further screening was performed after carefully reading the full manuscript.	Step III: appraisal
Snowball	Additional trials were found checking the citations of the included trials.	Step II–III: retrieval and appraisal
Data extraction	Number of events/total and mean +/− standard deviation were collected from each sty arm.	Step IV: synthesis
Literature re-check	A literature search was repeated to find new eligible trials since the initial search.	Step II–IV: retrieval, appraisal, synthesis
Analysis	Data were statistically combined from all the included trials.	Step IV: synthesis
Manuscript draft and editing	The final paper was written following the PRISMA checklist.	Step V: write-up
